# Extraction of BoNT/A, /B, /E, and /F with a Single, High Affinity Monoclonal Antibody for Detection of Botulinum Neurotoxin by Endopep-MS

**DOI:** 10.1371/journal.pone.0012237

**Published:** 2010-08-17

**Authors:** Suzanne R. Kalb, Consuelo Garcia-Rodriguez, Jianlong Lou, Jakub Baudys, Theresa J. Smith, James D. Marks, Leonard A. Smith, James L. Pirkle, John R. Barr

**Affiliations:** 1 Division of Laboratory Sciences, National Center for Environmental Health, Centers for Disease Control and Prevention, Atlanta, Georgia, United States of America; 2 Department of Anesthesia and Pharmaceutical Chemistry, University of California San Francisco, San Francisco, California, United States of America; 3 Battelle Memorial Institute at the Centers for Disease Control and Prevention, Atlanta, Georgia, United States of America; 4 Integrated Toxicology, United States Army Medical Research Institute of Infectious Diseases (USAMRIID), Ft. Detrick, Maryland, United States of America; University of California Merced, United States of America

## Abstract

Botulinum neurotoxins (BoNTs) are extremely potent toxins that are capable of causing respiratory failure leading to long-term intensive care or death. The best treatment for botulism includes serotype-specific antitoxins, which are most effective when administered early in the course of the intoxication. Early confirmation of human exposure to any serotype of BoNT is an important public health goal. In previous work, we focused on developing Endopep-MS, a mass spectrometry-based endopeptidase method for detecting and differentiating the seven serotypes (BoNT/A-G) in buffer and BoNT/A, /B, /E, and /F (the four serotypes that commonly affect humans) in clinical samples. We have previously reported the success of antibody-capture to purify and concentrate BoNTs from complex matrices, such as clinical samples. However, to check for any one of the four serotypes of BoNT/A, /B, /E, or /F, each sample is split into 4 aliquots, and tested for the specific serotypes separately. The discovery of a unique monoclonal antibody that recognizes all four serotypes of BoNT/A, /B, /E and /F allows us to perform simultaneous detection of all of them. When applied in conjunction with the Endopep-MS assay, the detection limit for each serotype of BoNT with this multi-specific monoclonal antibody is similar to that obtained when using other serotype-specific antibodies.

## Introduction

Botulinum neurotoxins (BoNTs) are protein neurotoxins produced by some species of the genus *Clostridium*, in particular, *Clostridium botulinum*, *C. butyricum*, *C. baratii*, *and C. argentinense*. Intoxication with any one of the seven serotypes of BoNT (A–G) causes botulism, a disease that is naturally contracted by either ingestion of food containing the toxin [1], [2], colonization of the bacteria in the gastrointestinal tract of infants or immunocompromised individuals, inhalation of the toxin, or contact of the bacterium with a wound [1]. Due to its extreme toxicity, global availability, and ease of preparation, it is considered a likely agent for bioterrorism [3]. The best therapeutic product for botulism involves administration of therapeutic immunoglobulin which is most effective when administered within 24 hr of exposure [1]. However, some currently commercially-available antitoxins are serotype-specific, so those products will not protect or save a patient if botulism is caused by any of the other serotypes. Therefore, the earliest determination of any specific serotype exposure to BoNT is critical before choosing the right antitoxin for treating a patient.

Previously, we reported the development of an assay for BoNT detection and serotype differentiation termed the Endopep-MS method [4]–[10]. This method detects all four BoNT serotypes known to affect humans, BoNT/A, /B, /E, and /F. Briefly, this method involves incubating BoNT with a peptide substrate that mimics the natural target of BoNT. Each BoNT cleaves its peptide substrate in a specific location, which is different for each of the BoNT serotypes tested [2], [4], [6], [9]. The reaction mixture is then introduced into a mass spectrometer, which detects and accurately reports the mass of any peptides within the mixture. Detecting the peptide cleavage products corresponding to their specific toxin-dependent location indicates the presence of a particular BoNT serotype. Historically, mouse bioassays have been the most commonly used method to detect BoNT [11], but as previously demonstrated in several publications [4]–[6], [9], the Endopep-MS method can more rapidly detect BoNT at levels comparable to or lower than levels detected with mouse bioassays.

As previously reported, Endopep-MS is highly effective in identifying BoNT/A, /B, /E, and /F, the four serotypes that commonly affect humans, in clinical samples. This method uses an antibody affinity concentration/purification step prior to reacting with the substrate [6]–[10]. Both polyclonal and monoclonal antibodies to BoNT/A, /B, /E, and /F were evaluated for use with the assay, and whenever possible, monoclonal antibodies were chosen in the assay due partly to their ability to recognize specific epitopes on the toxin, ensuring that they recognize the same epitopes with the same affinity during different test batches. Polyclonal antibodies can also interfere with the enzymatic activity of BoNT as measured by Endopep-MS [6], [10], because Endopep-MS detects the presence of BoNT specifically by measuring the activity of the neurotoxin.

Because the antibodies used in the affinity concentration/purification step are serotype-specific, in order to test for the presence of four serotypes, a sample must either be split into four aliquots or the same sample must be extracted four times, once with each serotype-specific antibody. Neither of these steps is ideal, since they negatively impact the assay in terms of sample volume requirements or total analysis time used, as each extraction step is approximately 1 hr. Therefore, the use of a single antibody which can extract all four serotypes of BoNT at once would be beneficial in an assay to rapidly detect BoNT. Here, we report the use of a unique high-affinity monoclonal antibody, 4E17.1, in the Endopep-MS assay for detection of all available subtypes of BoNT/A, /B, /E, and /F. This multi-specific cross reactive antibody allows for the simultaneous detection of BoNT/A, /B, /E,and /F by Endopep-MS without requiring any additional time for multiple toxin extractions, and it produces limits of detection which are similar to those obtained with serotype-specific monoclonal antibodies.

## Materials and Methods

### Ethics Statement

Studies using mice were conducted at USAMRIID, whose ethics committee specifically approved this study. Studies were performed under an approved IACUC protocol in compliance with the Animal Welfare Act and other federal statutes and regulations relating to animals and experiments involving animals, and adhere to principles stated in the Guide for the Care and Use of Laboratory Animals, National Research Council, 1996. The facility where this research was conducted is fully accredited by the Association for Assessment and Accreditation of Laboratory Animal Care International.

### Materials

Botulinum neurotoxin is very toxic and therefore requires appropriate safety measures. All neurotoxins were handled in a level 2 biosafety cabinet equipped with HEPA filters. Commercially purified complex toxins BoNT/A1, BoNT/A2, BoNT/B1, BoNT/C, BoNT/E3, protBoNT/F, and BoNT/G were purchased from Metabiologics (Madison, WI). Tetanus toxin (TeNT) was purchased from List Biological Laboratories, Inc. (Campbell, CA). Tetanus toxin antibody (TetE3) was purchased from Abcam (Cambridge, MA). Polyclonal rabbit IgGs for BoNT/E and BoNT/F were purchased from Metabiologics. Monoclonal antibodies CR2, RAZ1, and B12.1 were provided by Professor James Marks of the University of California, San Francisco. Dynabeads® Protein G were purchased from Invitrogen (Carlsbad, CA) at 1.3 g/cm^3^ in phosphate buffered saline (PBS), pH 7.4, containing 0.1% Tween®-20 and 0.02% sodium azide. Sequencing grade modified trypsin at 0.5 mg/mL in 50 mM acetic acid was purchased from Promega (Madison, WI). All chemicals were from Sigma-Aldrich (St. Louis, MO) except where indicated. Peptide substrates were synthesized by Los Alamos National Laboratory (Los Alamos, NM, Table 1).

**Table 1:** Amino acid sequence of peptides used in and produced by the Endopep-MS method.

Peptide	Sequence	*m/z* observed
BoNT/A substrate	Biotin-KGSNRTRIDQGN**QR**ATRXLGGK-Biotin	2878.7
BoNT/A NT product	Biotin-KGSNRTRIDQGNQ	1699.9
BoNT/A CT product	RATRXLGGK-Biotin	1197.8
BoNT/B substrate	LSELDDRADALQAGAS**QF**ETSAAKLKRKYWWKNLK	4024.7
BoNT/B NT product	LSELDDRADALQAGASQ	1759.9
BoNT/B CT product	FETSAAKLKRKYWWKNLK	2283.4
BoNT/E substrate	IIGNLRHMALDMGNEIDTQNRQID**RI**MEKADSNKT	4041.1
BoNT/E NT product	IIGNLRHMALDMGNEIDTQNRQIDR	2923.6
BoNT/E CT product	IMEKADSNKT	1136.6
BoNT/D and /F substrate	LQQTQAQVDEVVDIMRVNVDKVLERD**QKL**SELDDRADAL	4497.5
BoNT/D NT product	LQQTQAQVDEVVDIMRVNVDKVLERDQK	3296.9
BoNT/D CT product	LSELDDRADAL	1217.7
BoNT/F NT product	LQQTQAQVDEVVDIMRVNVDKVLERDQ	3167.8
BoNT/F CT product	KLSELDDRADAL	1345.2

The observed *m/z* of each peptide is also included.

### Preparation of 4E17.1 Antibodies

Monoclonal antibody (mAb) 4E17.1 was first selected and engineered as a single chain fragment of variable domain (scFv) antibody using yeast display technology. The original antibody gene was selected from material of human volunteers immunized with pentavalent BoNT toxoid using toxin of the BoNT/E3 subtype [12]–[15]. The scFv antibody was converted into full length IgG by subcloning the V-gene into a human IgG1 expression vector with IgG1/kappa constant regions and expressed in Chinese Hamster Ovary cells. mAb 4E17.1 was purified to greater than 90% homogeneity by protein G affinity chromatography from the cell culture supernatant, and then buffer exchanged into phosphate buffered saline (PBS) at a concentration of 1 mg/ml as previously described [14]–[16].

### Epitiope Mapping of 4E17.1 Antibodies

As discussed in greater detail in work by Garcia-Rodriguez, et.al. [17] and similar to work reported by Garcia-Rodriguez, et.al. [18] and Levy, et.al. [19], yeast displayed BoNT H_C_, H_N_, and L_C_ domains were stained with 4E17.1 IgG. Because 4E17.1 was shown to bind only to the H_N_ domain, the H_N_ sequences of the 7 BoNT serotypes were aligned and inspected for regions which were conserved in BoNT/A, /B, E, and F. 750–758 of BoNT/A was identified as conserved between those serotypes. Single alanine mutants were created for that region and displayed on the surface of yeast. The affinity for 4E17.1 of those mutants was measured and residues Y750, Y753, E756, and E757 within the translocation domain of BoNT/A were found to play an important role in 4E17.1 binding.

### Production of BoNT/A, /B, /D, /E, and /F culture supernatants

Crude culture supernatants representing various BoNT/A, /B, /D, /E, and /F subtypes (Table 2) were produced by incubating subcultures of each strain for 5 days at 30–35°C. After centrifugation, supernatants were removed and filtered through 0.22 µm filters. The filtered supernatants were tested for upper limits of toxicity, which indicated that the toxins were all present at concentrations of ≤10 µg/mL. Some of the preparations were titered to determine lethality in mLD_50_/mL.

**Table 2:** Sequence alignment of known BoNT subtypes in the range of Y750 to E757 of BoNT/A.

Toxin	Strain	Accession #	Amino acid sequence	K_D_ (pM)
BoNT/A1	Hall	AF488749	**Y** N Q **Y** T E **E E**	1.83
BoNT/A2	CDC 1436	EF028393	**Y** N Q **Y** T E **E E**	7.98
BoNT/A3	Loch Maree	DQ185900	**Y** N Q **Y** T E **E E**	4.5
BoNT/A4	Strain 657	EU341407	**Y** N Q **Y** T E **E E**	ND
BoNT/A5	H0 4402 065	EU679004	**Y** N Q **Y** T E **E E**	ND
BoNT/B1	Okra	AB232927	**Y** N I **Y** S E **K E**	43760
BoNT/B2	213B (ATCC 7949)	EF028395	**Y** N I **Y** S E **K E**	41330
BoNT/B3	CDC 795	EF028400	**Y** N I **Y** S E **K E**	ND
BoNT/B4	Eklund 17B	EF051570	**Y** N I **Y** S E **E E**	16.52
BoNT/B5	Strain 657	EF033130	**Y** N I **Y** S E **K E**	45210
BoNT/B6	Osaka05	AB302852	**Y** N I **Y** S E **K E**	ND
BoNT/C	Stockholm	X62389	**Y** K K **Y** S G **S D**	NB
BoNT/D	CB-16	S49407	**Y** K K **Y** S G **S D**	NB
BoNT/E1	German sprats (Hazen 35396)	AB082519	**Y** N S **Y** T L **E E**	730
BoNT/E2	CDC 5247	EF028404	**Y** N S **Y** T L **E E**	205
BoNT/E3	Alaska E43	EF028403	**Y** N S **Y** T L **E E**	241
BoNT/E4	BL5262	AB088207	**Y** N S **Y** T L **E E**	260
BoNT/E5	LCL155	AB037704	**Y** N S **Y** T L **E E**	ND
BoNT/E6	K35	AM695752	**Y** N S **Y** T L **E E**	ND
BoNT/F1	Langeland	GU213203	**Y** N N **Y** T S **D E**	65080
BoNT/F2	Strain 84	Y13631	**Y** N N **Y** T S **D E**	ND
BoNT/F3	CDC 54086	GU213218	**Y** N N **Y** T S **D E**	ND
BoNT/F4	CDC 54078	GU213214	**Y** N S **Y** T S **D E**	ND
BoNT/F5	CDC 54074	GU213211	**Y** N N **Y** T S **D E**	ND
BoNT/F6	Eklund 202F	M92906	**Y** N N **Y** T S **D E**	ND
BoNT/F7	Sullivan	HM746656	**Y** N N **Y** T L **D E**	ND
BoNT/G	113/30	X74162	**Y** N R **Y** S E **E D**	ND
TeNT	CN3911 (Harvard)	X06214	**Y** K I **Y** S G **P D**	ND

Residues that play an important role in 4E17.1 binding are bolded and underlined. Dissociation rates (K_D_) in pM of BoNT with mAb 4E17.1 are also listed. NB indicates no binding was observed and ND indicates that the K_D_ was not determined. Each BoNT is also identified by strain tested where appropriate. Equilibrium dissociation constant (K_D_) were measured by flow fluorimetry in a KinExA [17].

For toxin titrations, samples were diluted 2-fold in gelatin-phosphate buffer (0.2% gelatin, 0.4% sodium phosphate), pH 6.2. Female Crl∶CD-1 mice, 16–22 g on receipt (Charles River Laboratories, Raleigh, NC) were injected i.p. with total volumes of 0.1 mL diluted toxin. Mice were observed for 5 days, survivors were tallied, and the results were analyzed using probit analysis (SPSS, Chicago, IL). The final LD_50_/mL of the preparation was calculated by dividing the initial dilution used in the assays by the probit result. Titrations were done in duplicate and averaged to obtain the working titers for each toxin. Duplicate titrations were also done on commercially obtained toxins (Metabiologics, Madison, WI; Wako Biologicals, Richmond, VA) using their stated toxicity as the initial dilution, and the titers were averaged to obtain their working titer.

### Neurotoxin Extraction

Monoclonal antibody 4E17.1 was immobilized and crosslinked to the Dynabeads® Protein G as described in the manufacturer's protocol using 40 µg of antibody diluted into 500 µL of PBS for every 100 µ L of Dynabeads® Protein G. Cross-linked IgG-coated Dynabeads® were stored in PBS-Tween buffer (PBS with 0.05% Tween®-20) at 4°C for up to 12 weeks. For the BoNT extraction assay, an aliquot of 20 µL of antibody-coated beads was mixed for 1 hr with a solution of 5–100 µL of each culture supernatant, which is mixed with 495 µL of phosphate buffered saline with 0.01% Tween (PBST) buffer. After mixing for 1 hr with constant agitation at room temperature, the beads were washed twice in 1 mL each of PBST and then washed once in 100 µL of water. For limit of detection tests, BoNT at varying levels was spiked into an aliquot of 500 µL of PBST. Negative controls consisted of PBST with no spiked toxin extracted using the above protocol. For TeNT work, 5 µg of TeNT was spiked into 50 µL of PBST with the remainder of the extraction protocol as above. For simultaneous multiple serotype BoNT detection, 100 mLD_50_ of BoNT/A, /B, /E, and /F were spiked into 500 µL of serum mixed with 50 µL of 10× PBST.

### Endopep-MS reaction

The reaction was performed as previously described [4]–[10] with a few modifications. In all cases, the final reaction volume was 20 µL; the final concentration of the reaction buffer was 0.05 M Hepes (pH 7.3), 25 mM dithiothreitol, 20 µM ZnCl_2_, and 1 mg/mL bovine serum albumin; and the final concentration of the peptide substrate was 50 pmol/µL, with peptide sequences listed in Table 1. All samples were incubated at 37°C for 4 hrs.

### MS Detection of Endopep-MS reaction

A 2 µL aliquot of each reaction supernatant was mixed with 18 µL of matrix solution consisting of alpha-cyano-4-hydroxy cinnamic acid (CHCA) at 5 mg/mL in 50% acetonitrile, 0.1% trifluoroacetic acid (TFA), and 1 mM ammonium citrate. A 0.5 µL aliquot of the resulting milieu was pipeted onto a 192-spot matrix-assisted laser desorption/ionization (MALDI) plate (Applied Biosystems, Framingham, MA). Mass spectra of each spot were obtained by scanning from 1100 to 4800 *m/z* in MS-positive ion reflector mode on an Applied Biosystems 4800 Proteomics Analyzer (Framingham, MA). The instrument used a Nd-YAG laser at 355 nm, and each spectrum was an average of 2400 laser shots.

### Tryptic Digest and Mass Spectrometric Analysis of TeNT

Following bead extraction of spiked TeNT from PBST buffer, the beads were reconstituted in 10 µL of 50 mM ammonium bicarbonate, pH = 7.5 and 2 µL of stock trypsin. 2 µL of 500 mM ammonium bicarbonate, pH = 7.5 and 2 µL of stock trypsin were also added to 20 µL of the extraction supernatant. These mixtures were digested overnight at 37°C. Following digestion, 1 µL of 10% TFA was added to both mixtures. The supernatant was then removed from the beads for analysis. 5 µL of this supernatant and the extraction supernatant mixture were analyzed by LC-MS/MS for amino acid sequence as reported by Kalb, et.al. [7].

## Results

### Monoclonal Antibody 4E17.1 Binds Multiple Subtypes of BoNT/A

Monoclonal antibody (mAb) 4E17.1 interacts with residues Y750, Y753, E756, and E757 within the translocation domain of BoNT [17]. A sequence alignment (Table 2) of the five currently-recognized subtypes of BoNT/A shows that all subtypes contain these same residues; therefore, mAb 4E17.1 should theoretically bind all known subtypes of BoNT/A. Protein G beads were coated with mAb 4E17.1 and used to extract toxins of BoNT/A1, /A2, /A3, and /A4 from individual culture supernatants.

**Figure 1:**
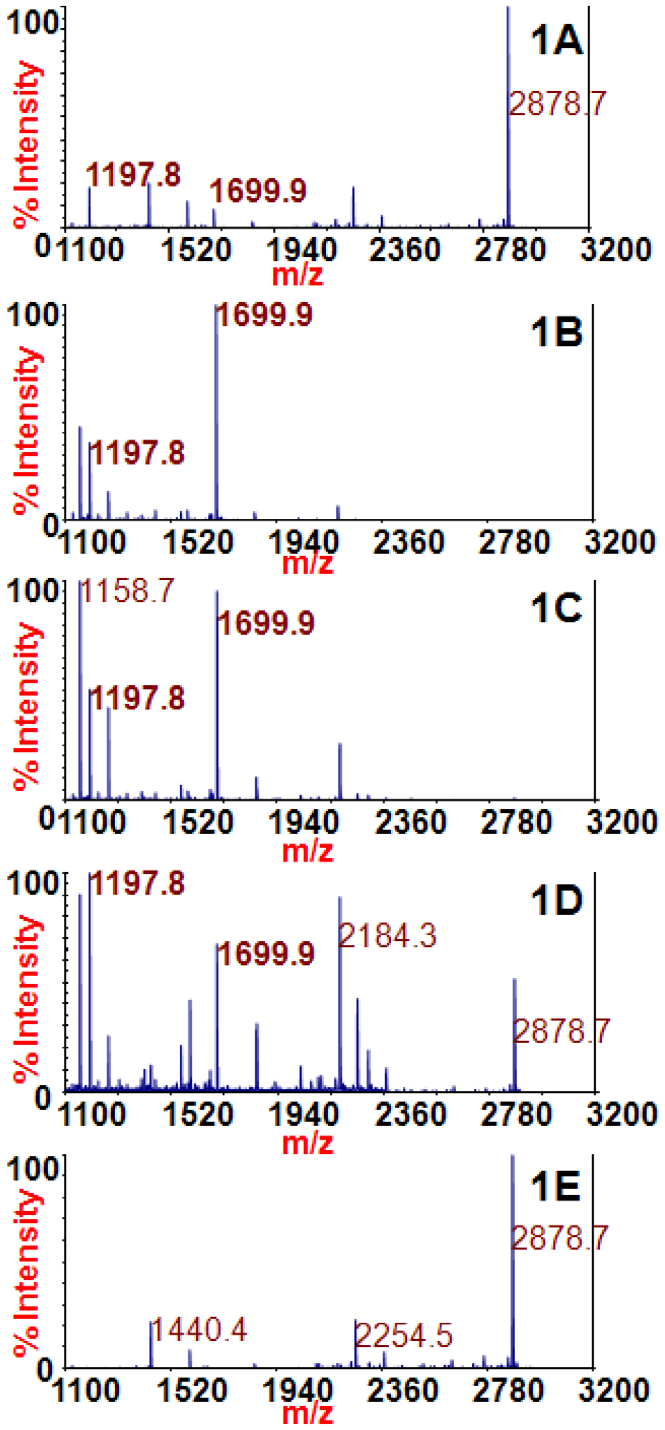
Mass spectra of the Endopep-MS BoNT/A reactions with /A1 (1A), /A2 (1B), /A3 (1C), /A4 (1D), or no toxin (1E) extracted using the 4E17.1 antibody-coated beads. The peptide cleavage products indicating BoNT/A are *m/z* 1197.8 and 1699.9; the peptide substrate is present at *m/z* 2878.7.

Figure 1 includes the mass spectra obtained from the reactions of the extracted BoNT/A toxins. In the presence of BoNT/A, the peptide substrate at *m/z* 2878.7 is cleaved by the toxin to produce peaks at *m/z* 1197.8 and 1699.9 (identities listed in Table 1). Hence, peaks at 1197.8 and 1699.9 are evidence for the presence of BoNT/A, as BoNT/A1, /A2, /A3, and /A4 are all known to cleave this peptide substrate [9]. The mass spectra in Figures 1A–1D all contain peaks at *m/z* 1197.8 and 1699.9 and therefore those reactions contain BoNT/A toxin. Figure 1E is a negative control of blank culture supernatant medium that does not contain toxin; therefore there are no peaks at *m/z* 1197.8 and 1699.9. These spectra illustrate that the affinity of mAb 4E17.1 for BoNT/A1, /A2, /A3, or /A4 is sufficient for the effective extraction and detection of all BoNT/A subtypes via Endopep-MS.

### Monoclonal Antibody 4E17.1 Binds Multiple Subtypes of BoNT/B, /E, and /F

After determining that mAb 4E17.1 could be used successfully to extract and detect all subtypes tested of BoNT/A via Endopep-MS, we investigated the use of this antibody to detect BoNT/B as well. The sequences of BoNT/B subtypes within the 4E17.1 epitope are listed in Table 2 and indicate that for most subtypes, 3 of the 4 amino acids essential for 4E17.1 binding (Y750, Y753, and E757) are present. BoNT/B4 contains all 4 amino acids essential for binding; Y750, Y753, E756, and E757.

**Figure 2:**
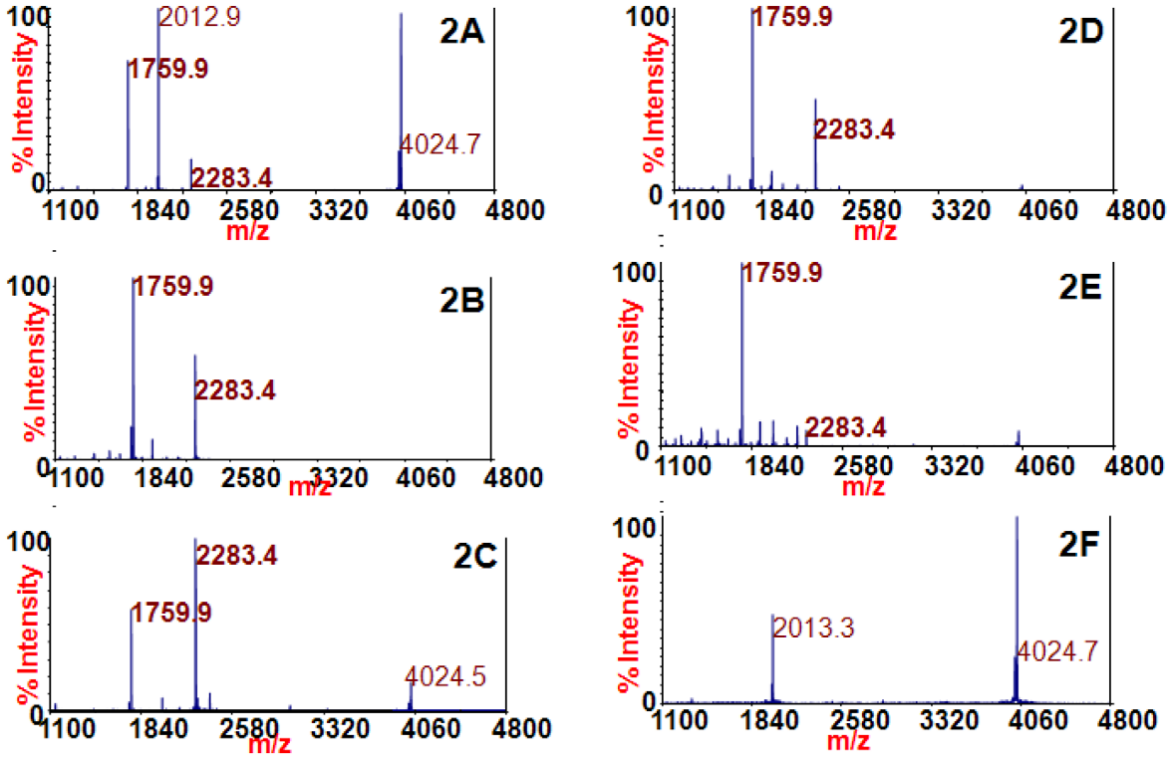
Mass spectra of the Endopep-MS BoNT/B reactions with /B1 (2A), /B2 (2B), /B3 (2C), /B4 (2D), /B5 (2E), or no toxin (2F) extracted using the 4E17.1 antibody-coated beads. The peptide cleavage products indicating BoNT/B are *m/z* 1759.9 and 2283.4 and the peptide substrate is present at *m/z* 4024.7.

Figure 2 shows the mass spectra obtained from the reactions of the extracted BoNT/B toxins with mAb 4E17.1. The peptide substrate at *m/z* 4024.7 is cleaved by BoNT/B to produce peaks at *m/z* 1759.9 and 2283.4 (identities listed in Table 1), and BoNT/B1, /B2, /B4, and /B5 are all known to cleave this peptide substrate [9]. Similar to the procedures used for BoNT/A extraction, BoNT/B1, /B2, /B3, /B4, and /B5 were extracted separately with beads coated with mAb 4E17.1, and peptide substrate in reaction buffer was added to those beads. The mass spectra in Figures 2A–2E all contain peaks at *m/z* 1759.9 and 2283.4, indicating that those reactions contained active BoNT/B toxin. Figure 2F is a negative control of blank culture supernatant medium which does not contain toxin; therefore there are no peaks at *m/z* 1759.9 and 2283.4. These spectra demonstrate that monoclonal antibody 4E17.1 has adequate affinity for BoNT/B1, /B2, /B3, /B4, and /B5 for this antibody to be used to extract and detect BoNT/B via Endopep-MS. Additionally, this is the first report of BoNT/B3 detection with Endopep-MS.

**Figure 3:**
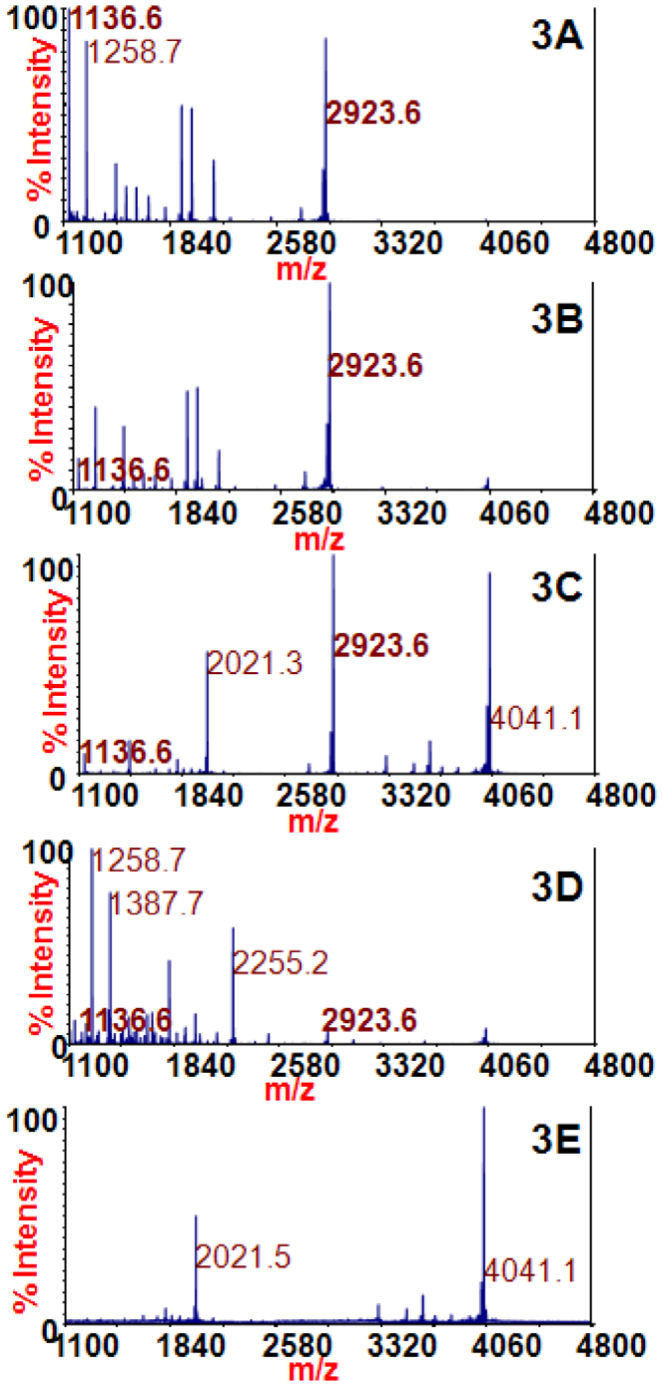
Mass spectra of the Endopep-MS BoNT/E reactions with /E1 (3A), /E2 (3B), /E3 (3C), /E4 (3D), or no toxin (3E) extracted using the 4E17.1 antibody-coated beads. The peptide cleavage products indicating BoNT/E are *m/z* 1136.6 and 2923.6 and the peptide substrate is present at *m/z* 4041.1.

Because the amino acid sequences of all BoNT/E subtypes contain all four amino acids necessary for optimum 4E17.1 binding, it was expected that the 4E17.1 antibody should be able to bind and extract all BoNT/E subtypes. Beads coated with 4E17.1 were therefore used to extract BoNT/E subtypes from culture supernatant media. Figure 3 provides the mass spectra obtained from the reaction of extracted BoNT/E1, /E2, /E3, and /E4 with the peptide substrate at *m/z* 4041.1. This peptide is cleaved by BoNT/E to produce cleavage products at *m/z* 1136.6 and 2923.6 (identities listed in Table 1), and subtypes BoNT/E1,/E2, /E3, and /E4 are all known to cleave this peptide substrate [9]. The mass spectra in figures 3A–3D all contain peaks at *m/z* 1136.6 and 2923.6, and therefore all contain BoNT/E toxin. The negative control in 3E does not contain BoNT/E toxin; hence, the peaks at *m/z* 1136.6 and 2923.6 are absent. These spectra illustrate that the 4E17.1 antibody can be utilized to extract BoNT/E1, /E2, /E3, and /E4 for detection via Endopep-MS.

**Figure 4:**
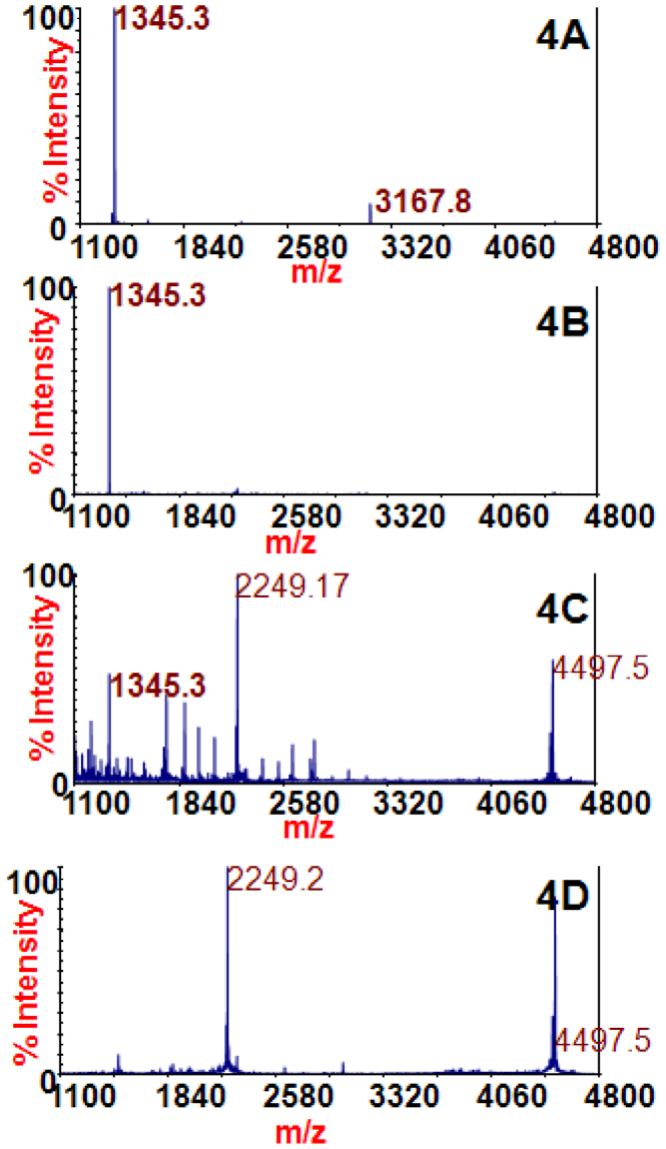
Mass spectra of the Endopep-MS BoNT/F reactions with /F1 (4A), /F6 (4B), /F2 (4C), or no toxin (4D) extracted using the 4E17.1 antibody-coated beads. The peptide cleavage products indicating BoNT/F are *m/z* 1345.2 and 3167.8 and the peptide substrate is present at *m/z* 4497.5.

The amino acid sequences of the 7 BoNT/F subtypes contain Y750, Y753, and E757, three of the four amino acids determined to be important in 4E17.1 binding. The BoNT/B subtypes also contain these three amino acids, and it has already been shown that the BoNT/B subtypes recognize 4E17.1, so it was expected that the four subtypes of BoNT/F would also recognize 4E17.1. Figure 4 shows the mass spectra from the reaction of extracted BoNT/F1 (4A), BoNT/F6 (4B), and BoNT/F2 (4C) with the peptide substrate at *m/z* 4497.5. These subtypes of BoNT/F cleave this peptide substrate into cleavage products at *m/z* 1345.2 and 3167.8 (identities listed in Table 1), and those cleavage products are present in Figures 4A–4C, proving that the 4E17.1 antibody can be used to extract those BoNT/F subtypes and detect them via Endopep-MS. Figure 4D is the spectrum of blank culture supernatant medium extracted with the 4E17.1 antibody. It does not contain the cleavage products at *m/z* 1345.3 or 3167.8. BoNT/F from *C. baratii* (BoNT/F7) also binds this antibody, but requires a different peptide substrate to detect the toxin via its activity and will be addressed in a separate publication.

### Monoclonal Antibody 4E17.1 Does Not Bind BoNT/C and /D Effectively

BoNT/C and /D both contain Y750 and Y753, but not E756 or E757 (Table 2). Similar to the procedures used for BoNT/A or BoNT/B extraction, mAb 4E17.1 was used to extract BoNT/C and /D, and the BoNT bead extracts were tested for activity via Endopep-MS. Toxin levels below 20,000 mouse LD_50_ (mLD_50_) of BoNT/C and 2,000 mLD_50_ of BoNT/D did not yield any positive results by mass spectrometry, indicating that the toxin's activity is either inhibited by the 4E17.1 antibody or that the antibody is inefficient at toxin extraction. As a comparison, samples containing less than 1 mLD_50_ of BoNT/A, /B, /E, or /F were positive following extraction with monoclonal antibody 4E17.1. The dissociation rate (K_D_) for binding of 4E17.1 to BoNT/C and /D [17] was so poor that it could not be measured (Table 2). This effects the ability of this antibody to effectively bind BoNT/C or /D, making it inefficient at toxin extraction.

**Figure 5:**
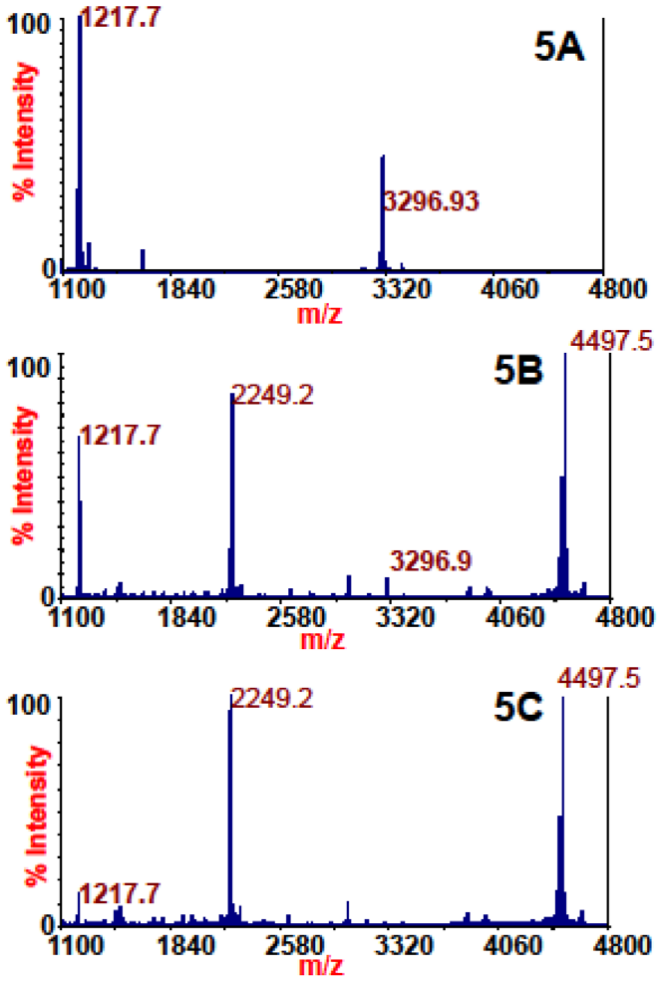
Mass spectra of the Endopep-MS BoNT/D reactions with no antibody extraction (5A), extraction with monoclonal antibody 4E17.1 (5B), or extraction with monoclonal antibody CR2 (5C). The peptide cleavage products indicating BoNT/D are *m/z* 1217.7 and 3296.9 and the peptide substrate is present at *m/z* 4497.5.

Figure 5A shows a mass spectrum of 2000 mLD_50_ of BoNT/D with no antibody extraction. Peaks at *m/z* 1217.7 and 3296.9 (identities listed in Table 1) indicate the presence of BoNT/D in a sample. The substrate at *m/z* 4497.5 is not visible as it has been completely consumed by the toxin. This is contrasted with Figure 5B, which is the mass spectrum of 2000 mLD50 of BoNT/D extracted with beads coated with monoclonal antibody 4E17.1. The cleavage products at *m/z* 1217.7 and 3296.9 are both visible in this spectrum, but the dominant peak in this spectrum is the singly-charged substrate at *m/z* 4497.5 and the doubly-charged substrate peak at *m/z* 2249.1. Conversion of the substrate to the cleavage products is vastly diminished in this spectrum as compared to the spectrum in Figure 5A. This is due to the lower level of toxin in the sample as most of the toxin remains behind following extraction.

Additionally, some of this toxin activity can be explained by non-specific binding of the toxin to the beads. This is illustrated in Figure 5C, which is the mass spectrum of 2000 mLD_50_ of BoNT/D extracted with beads coated with monoclonal antibody CR2, an antibody specific for BoNT/A, but not cross reactive with BoNT/D. One cleavage product at *m/z* 1217.7 is visible in this spectrum, but the dominant peaks in this spectrum are the singly-charged and doubly-charged substrate peaks at *m/z* 4497.5 and 2249.1 respectively. Presence of the cleavage product at *m/z* 1217.7 indicates the presence of a low level of BoNT/D on the beads due to non-specific binding of the toxin to the beads.

### Monoclonal Antibody 4E17.1 Does Not Bind BoNT/G and Tetanus Toxin

Tetanus toxin (TeNT) contains Y750 and Y753, but not E756 or E757 (Table 2). BoNT/G contains Y750, Y753, and E756, but not E757. Similar to the procedures for BoNT/A extraction, mAb 4E17.1 was used to extract BoNT/G, and the BoNT bead extracts were tested for activity via Endopep-MS. This did not yield any positive result by mass spectrometry, indicating that the antibody is inefficient at toxin extraction. We have not developed a peptide-based activity assay for TeNT, and we therefore could not test for the activity of TeNT upon a peptide substrate as evidence for its binding to monoclonal antibody 4E17.1. Therefore, beads coated with monoclonal antibody 4E17.1 or with a commercially-available monoclonal antibody to tetanus toxin were used to extract TeNT. The beads were then digested with trypsin and the tryptic fragments were analyzed by LC-MS/MS.

There were several peptides which originated from TeNT in the digest of the beads which used anti-TeNT for extraction, but these peptides were not present in the digested beads which used anti-4E17.1 for extraction (data not shown). Furthermore, a digest of the supernatant consisting of material left behind after the extraction of TeNT with 4E17.1 coated beads demonstrated that TeNT remained behind in solution following extraction with 4E17.1 coated beads (data not shown).

### Monoclonal Antibody 4E17.1 Binds Different BoNT Serotypes Simultaneously

**Figure 6:**
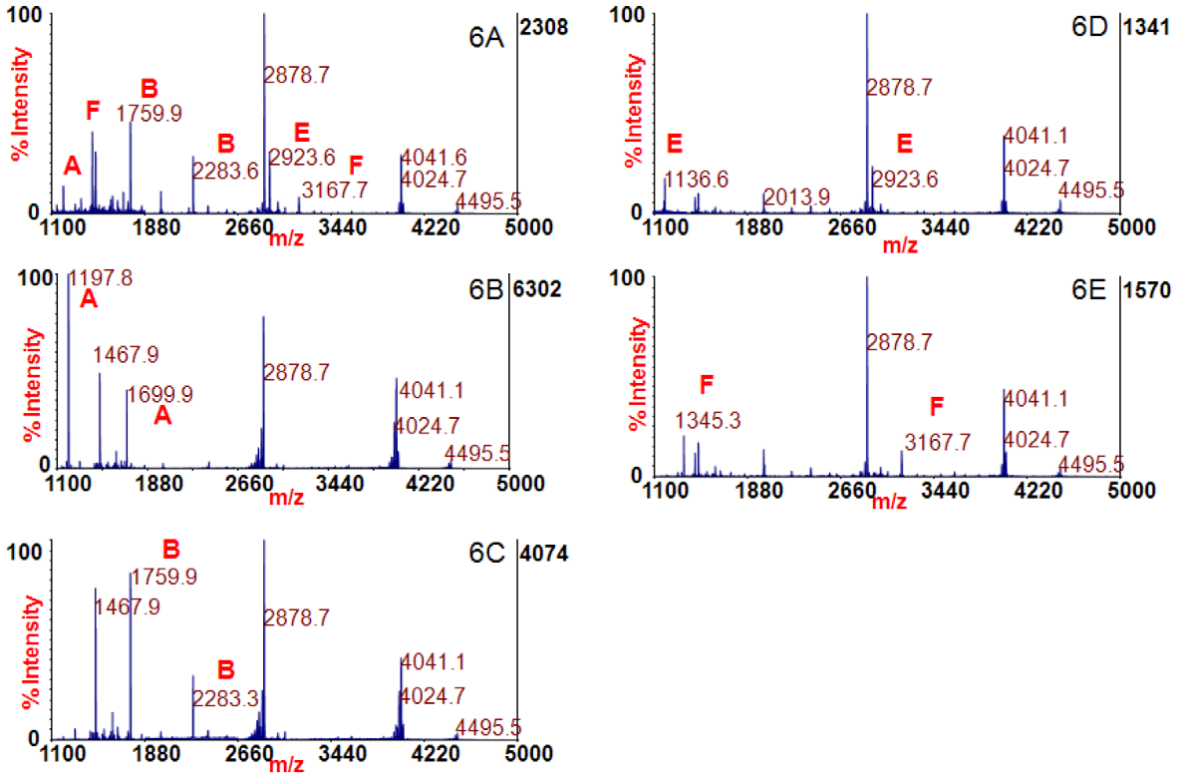
Mass spectrum of the Endopep-MS reaction of the simultaneous extraction of BoNT/A, /B, /E, and /F with monoclonal antibody 4E17.1 (6A) or the single extraction of BoNT/A (6B), /B(6C), /E (6D), or /F (6E) with serotype-specific antibodies. Cleavage products indicating those four BoNTs are marked.

Monoclonal antibody 4E17.1 has been shown to bind separately multiple subtypes of BoNT/A, /B, /E, and /F; therefore, it was assumed that this antibody could bind multiple subtypes of BoNT/A, /B, /E, and /F simultaneously. Beads coated with 4E17.1 antibody were used to extract BoNT/A, /B, /E, and /F spiked into a single sample. The beads were then added to reaction buffer with the four peptide substrates used to detect BoNT/A, /B, /E, and /F. Figure 6A shows the mass spectrum from that reaction. Peaks at *m/z* 1197.8 and 1699.9 indicate the presence of BoNT/A; 1759.9 and 2283.4 are markers for BoNT/B; 1136.6 and 2923.6 for BoNT/E; and 1345.2 and 3167.8 indicate BoNT/F, and the identities of all cleavage products are listed in Table 1. The presence of all these peaks demonstrates that monoclonal antibody 4E17.1 can be used to extract BoNT/A, /B, /E, and /F from a single sample and that all four serotypes can be detected at once.

Although the toxins were present in an equimolar ratio in this case, we also experimented with each individual toxin present at levels up to 1000 times the others. Those results (data not shown) indicate that we can still detect all four toxins in that case. Additionally, serotype specific antibodies were also used to extract BoNT/A, /B, /E, and /F spiked into serum, and those results are depicted in Figures 6B, 6C, 6D, and 6E. These results demonstrate that all four serotypes of BoNT can be detected individually using 4 times the sample volume.

**Figure 7:**
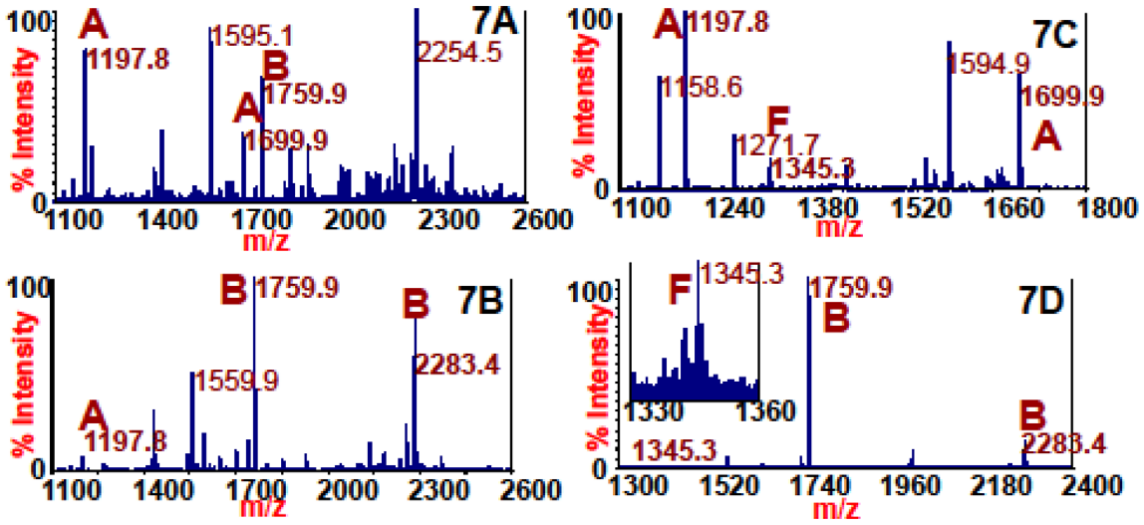
Mass spectra from the reaction of peptide substrates with 4E17.1 coated beads used to extract A2b (7A), Ba4 (7B), Af (7C), and Bf (7D). Peaks at *m/z* 1197.8 and 1699.9 indicate BoNT/A, peaks at *m/z* 1759.9 and 2283.4 indicate BoNT/B, and peaks at *m/z* 1345.2 and 3167.8 result from BoNT/F activity.

Under natural conditions, it is unlikely that a single sample would contain four serotypes of BoNT; however, there are some cases when a single sample contains more than one serotype. Several bivalent strains exist, known as A2b, Ba4, Af, and Bf, in which the bacterium produces more than one serotype of BoNT under certain conditions. Figure 7 shows that monoclonal antibody 4E17.1 can be used to detect these bivalent strains, as they are the mass spectra acquired from the reaction of peptide substrates with 4E17.1 coated beads used to extract A2b (7A), Ba4 (7B), Af (7C), and Bf (7D). In Figure 7A, peaks at *m/z* 1197.8 and 1699.9 illustrate the presence of BoNT/A, and the peak at *m/z* 1759.9 indicates BoNT/B. In Figure 7B, the peak at *m/z* 1197.8 shows BoNT/A, and peaks at *m/z* 1759.9 and 2283.4 result from BoNT/B activity. In Figure 7C, the peak at *m/z* 1197.8 indicates BoNT/A, whereas the peak at *m/z* 1345.3 indicates BoNT/F. In Figure 7D, peaks at *m/z* 1759.9 and 2283.4 indicate BoNT/B, and the peak at *m/z* 1345.2 results from BoNT/F activity.

### Limit of Detection of BoNT with Monoclonal Antibody 4E17.1 in Endopep-MS

The Endopep-MS assay detects BoNT via its activity, and it uses antibody extraction to isolate and concentrate the toxin prior to analysis. Some antibodies inhibit the enzymatic activity of the toxin and therefore increase the limit of detection (LOD) of BoNT. We therefore wanted to determine the LOD of BoNT/A, BoNT/B, BoNT/E, and BoNT/F using mAb 4E17.1 for extraction of those toxins from buffer. Using commercially-available, mouse-titered BoNT/A, /B, /E, and /F, we determined that the LOD of those toxins spiked into 0.5 mL of PBST buffer are 1 mLD_50_ for BoNT/A, 0.05 mLD_50_ of BoNT/B, 0.1 mLD_50_ of BoNT/E, and 0.05 mLD_50_ of BoNT/F. These LOD are equivalent to those obtained with other serotype-specific antibodies used for toxin extraction, with the exception of BoNT/A, and the LOD in serum and milk are the same as in PBST buffer. The LOD of BoNT/A using a combination of the RAZ1 and CR2 antibodies engineered for high affinity to BoNT/A is 0.5 mLD_50_.

## Discussion

The different serotypes of BoNT are defined by their ability to be neutralized by specific antitoxins; therefore, it is unusual for one antibody to bind multiple serotypes of botulinum neurotoxin. Monoclonal antibody 4E17.1 binds the translocation domain of BoNT, and residues Y750, Y753, E756, and E757 of BoNT/A play important roles in antibody binding. These residues are fairly well conserved among all of the known botulinum neurotoxins; hence, a single monoclonal antibody could bind most of the known botulinum neurotoxins at the same epitopic location.

All currently known subtypes of BoNT/A contain residues Y750, Y753, E756, and E757. BoNT/A1-/A4 were tested for their ability to bind monoclonal antibody 4E17.1. To test for the presence or absence of the extracted toxin on the antibody-coated beads, we used Endopep-MS. BoNT/A1-/A4 were all efficiently extracted from buffer with antibody 4E17.1, and the beads from those extractions all demonstrated the presence of active BoNT/A, meaning that all four of those BoNT/A subtypes bind the 4E17.1 antibody. Although BoNT/A5 was unavailable for testing, it is expected that this serotype will also effectively interact with 4E17.1 on the basis of its amino acid sequence (Y750, Y753, E756, and E757) [20]. In fact, the amino acid sequence YNQYTEEE is identical in 63 BoNT/A strains.

Most subtypes of BoNT/B contain three of the four key amino acids in 4E17.1 binding; the exception to this is BoNT/B4, which contains all four amino acids. Five of the currently known subtypes of BoNT/B were available for testing, and all interacted with the 4E17.1 antibody. This interaction indicates that E756 is perhaps not as critical for binding as the other amino acids. E756 is a lysine in most of the BoNT/B serotypes, and a mutation from glutamic acid to lysine is not a conserved mutation. This non-conserved mutation may be responsible for the higher K_D_ associated with BoNT/B in general for this antibody (Table 2) [17]. Although BoNT/B6 was unavailable for testing, it is also expected to interact with 4E17.1 based on its amino acid sequence (Y750, Y753, K756, and E757) [21]. As with BoNT/A, this epitope is highly conserved among 64 BoNT/B strains as YNIYSEKE.

All currently known subtypes of BoNT/E contain all four key amino acids in 4E17.1 binding. Subtypes BoNT/E1–E4 were available for testing, and all four interacted with 4E17.1. Subtypes BoNT/E5 and /E6 were unavailable for testing, but based on the amino acid homology [22]–[23], both are expected to interact with 4E17.1. Additionally, the examination of 55 BoNT/E strains demonstrates that this epitope is identical as YNSYTLEE. All currently known subtypes of BoNT/F contain Y750, Y753, and E757, three of the four key amino acids in 4E17.1 binding. Four of the seven subtypes were available for testing, and all four interacted with 4E17.1. This is further evidence that E756 is perhaps not as critical for binding as the other residues, because E756 is an aspartic acid, a conserved mutation, in all serotypes of BoNT/F. Subtypes BoNT/F3, /F4, and /F5 were unavailable for testing, but based on the amino acid homology [24], all are expected to interact with 4E17.1. Furthermore, 60 strains of BoNT/F have this identical epitope YNNYTSDE.

BoNT/C, /D, /G, and TeNT all contain Y750 and Y753, two of the amino acids known to be important in 4E17.1 binding. However, upon testing, it was determined that these toxins do not efficiently interact with the 4E17.1 antibody. Hence, these data show that a minimum of three of the four critical amino acids, Y750, Y753, and E757, are required for efficient binding. Furthermore, BoNT/G contains three of the four key amino acids in 4E17.1 binding including Y750, Y753 and E756, but it does not contain E757 and BoNT/G does not interact with 4E17.1. These additional data show that E756 is not critical for 4E17.1 interactions but that Y750, Y753, and especially E757 are required for efficient binding.

Endopep-MS was developed to detect the presence of BoNT in a clinical or food sample. An antibody affinity concentration/purification step prior to reaction with the substrate is critical to the success of the method when testing complex matrices such as serum or stool extracts. Prior to the discovery of the 4E17.1 antibody, a sample needed to either be split into 4 aliquots or extracted 4 separate times to test for the presence of four serotypes of BoNT. Either option negatively affected the Endopep-MS assay in terms of either sample volume requirements or time as each extraction step is approximately 1 hr. Therefore, the development of a single antibody which can be used to extract four serotypes of BoNT simultaneously, especially the four serotypes commonly involved in human botulism, is beneficial in the Endopep-MS assay to detect BoNT and would be beneficial in other assays which use antibody-affinity for BoNT detection.

We have tested the 4E17.1 antibody with spiked clinical and food samples such as serum, stool extracts, and milk and found that this antibody is particularly effective with serum and milk. For stool extracts with a severe protease composition, we have had recent success with 2M sodium chloride washes to remove excessive proteases from the antibody-coated beads (manuscript in preparation). In the presence of such extreme conditions, BoNT/B and /F in particular unfortunately are removed from the antibody-coated beads, due to the higher dissociation rates [17] listed in Table 2. Thus, the use of the 4E17.1 antibody may be limited to stool extract samples which contain fewer proteases, such as infant stools. Further studies employing real-world clinical specimens are planned when such samples become available to us. Nevertheless, as the LOD of BoNT/A, /B, /E, and /F are equivalent or near equivalent to those obtained with other serotype-specific antibodies used for toxin extraction, this new antibody offers the opportunity to test for any of those toxin types in an unknown sample.

The opinions, interpretations and recommendations are those of the authors and are not necessarily those of the Centers for Disease Control and Prevention or the US Army.
